# Biological control potential of entomopathogenic fungal strains against peach Fruit fly, *Bactrocera zonata* (Saunders) (Diptera: Tephritidae)

**DOI:** 10.7717/peerj.13316

**Published:** 2022-04-22

**Authors:** Ghulam Murtaza, Muhammad Naeem, Saba Manzoor, Hammad Ahmad Khan, Emad M. Eed, Waqar Majeed, Hussain Ahmed Makki, Uzma Ramzan, Umm E. Ummara

**Affiliations:** 1 Department of Entomology, College of Plant Protection, China Agricultural University, Beijing, China; 2 College of Life Science, Hebei Normal University, Shijiazhuang, China; 3 Department of Zoology, University of Sialkot, Sialkot, Pakistan; 4 Department of Zoology, Wildlife and Fisheries, University of Agriculture Faisalabad, Faisalabad, Pakistan; 5 Department of Clinical Laboratory Sciences, College of Applied Medical Sciences, Taif University, Taif, Saudi Arabia; 6 Department of Forestry, Range and Wildlife Management, The Islamia University Bahawalpur, Bahawalpur, Pakistan; 7 Institute of Zoology, University of the Punjab, Lahore, Pakistan

**Keywords:** *B. zonata*, Entomopathogenic fungi, Bioinsecticides, Mortality, Integrated pest management

## Abstract

The peachfruit fly, *Bactrocera zonata* (Saunders) is a polyphagous pest in nature, belonging to order, *Diptera* and their respective family is *Tephritidae*. It mostly feeds on different crops, vegetables and fruits. Different traditional chemical insecticides have been used to control this notorious pest. Excessive consumption of pesticides has become a major threat to the fresh fruits trade since many importing countries refused to accept the shipments due to public health and environmental concerns. There is a growing trend to control these pests using the most effective biological control methods and other preventive measures have been adopted for reducing their attacks. Fungal agents have been used as biological agents to manage the attack of different insects pest through biological means. The present study was conducted to assess the virulence of three entomopathogenic fungi, *Metarhizium anisopliae*, *Beauveria bassiana* and *Verticillium lecanii,* against *Bactrocera zonata* stages under different laboratory conditions. The results showed that *B. bassiana* and *M. anisopliae* were more effective in pathogenicity and potentially kill at all stages of *B. zonata* as compared to *V. lecanii*. The highest mortality rate for the third larval instar and the pupal stage were recorded after exposure to the 1 × 10^10^ conidia/ml concentrations, *B. bassiana,* with 68.67% and 89.67%, respectively. Adult *B. zonata* flies were the most susceptible to all entomopathogenic fungi. However, *M. anisopliae* was more virulent against *B. zonata* adult flies than *B. bassiana* and *V. lecanii* at 1 × 10^10^ conidial concentration. Therefore, the entomopathogenic fungi *B. bassiana* and *M. anisopliae* can be used as an cost effective bio-insecticide in the integrated pest management programs to control *B. zonata*. This study will be helpful to overcome this pest through biological control means.

## Introduction

The peach fruit fly *Bactrocera zonata* (Saunders) (Diptera: Tephritidae) usually attacks vegetables and fruits and is a significant polyphagous pest (El-Minshawy et al., 2018). Different plants have been affected through the attack of pests flies. They primarily attack fleshy fruits like mangos, apricots, guava, citrus and peaches (Hossain et al., 2017). *Bactrocera zonata* and *Tephritidae dacinae* are found in ASEAN countries and belong to a large genus of tephritid flies native to Asia, and their species are also found in different countries such as Srilanka, Thailand, Bangladesh, India, Egypt and Mauritius (El-Minshawy et al., 2018). Their high abundance occurs in the temperate seasons that increase the chances of an attack on different varieties of crops and vegetables (Mujica & Kroschel, 2011).

*B. zonata* has been considered a global economic pest. It is responsible for losses in Pakistan up to 89.50% and 10 to 20% in the Himalayan region (Hossain et al., 2017; Murtaza et al., 2021a). In different countries, *B. zonata* has been documented to cause 10–80% fruit losses in different crops, fruits and vegetables such as *Mangifera indica* L, peach, citrus and cereal crops under different ecological seasons (Ahmad & Begum, 2017). In India, its pest status is similar to that of *B. cucurbitae and B. dorsalis* which also affected *Mangifera indica* L., *Psidium guajava* L. and *Averrhoa carambola* L. (Hossain et al., 2017). It is the most dominating, destructive, and prolific fruit fly species and can cause a huge losses in crop production (Murtaza et al., 2021a).  According to the previously published research, in Pakistan, *B. zonata* is the most destructive, dominant, and abundant species of fruit fly infesting a huge spectrum of vegetables and fruits (Ahmad & Begum, 2017; Murtaza et al., 2021a). The control of this pest species will help to manage the damage of crops, fruits and vegetables ultimately benefiting in economics (Enkerlin, 2005).

*B. zonata* lay whitish, oblique, elongated eggs that are slightly rounded at the end and are 1.0 to 1.2 mm long. Eggs hatch in approximately 2–3 days and typically produce maggots. *B. zonata* has three larval instars called maggots: spirally head, three thoracic segments, and eight abdominal segments. Pupae have 11 segments and are barrel in shape and yellowish to brown-yellowish in color (Kwami, 2012). The female fruit flies pierce the skin of fruits by their ovipositor and lay eggs under the skin on soft and tender tissues. Eggs hatched inside the fruits and maggots start feeding and move toward the soft part of the fruits and watery fluid oozing out from the puncture side of the fruit (Murtaza et al., 2021b). Mostly, the use of pesticides is prevented in the control of this pest because pesticides residues are a big barrier to the fresh fruit trade since many ASEAN and well-developed countries like UK, Canada and other importing countries refuse to accept the shipments due to public health and environmental concerns. The bioaccumulation of the biopesticides in the living tissues can cause serious cellualr toxicties. Chemical pesticides have a harmful impact on the natural environment and negatively impact human health. The huge spread of agricultural pests poses a hazard to the food sector and human health. Therefore, there is a growing trend to control these pests using biological agents (Melo & Swarowsky, 2022). Different chemical-based fungicides and insecticides are used in the form of sprays in the soil applications for the controlling the attack of insects. These are chlorothalonil for controlling the broad spectrum infectious fungus and carbendazim for reducing the seasonal attcks of variety of insects (Bhat et al., 2019; Saritha & Prasad Tollamadugu, 2019).

For insect pests, the fungal agents are the most favorable application for biological control. In the soil, they are naturally present and easily come into contact with full-grown *B. zonata* larvae and pupae. *Beauveria bassiana* (Bals.) and *Metarhizium anisopliae* are the most imporatant entomopathogens of dipteran insects (Castillo et al., 2000; Destéfano et al., 2005; Ortu, Coceo & Dau, 2009; Boudjelida & Soltani, 2011). Entomopathogenic fungi infest the host insects by ingestion, respiration, and *via* the epidermis. Fungi produce mycelium to pierce the epicuticle and proceed into epithelial tissue to accomplish infection in the epidermis, which are among the most prevalent infestation mechanisms (Shah & Pell, 2003; Scholte et al., 2004). Anamorphic fungi such as *B. bassiana* and *M. anisopliae* propagate primarily through blastospores rather than hyphal growth (Chandler, 2017). These blastospores infiltrate vital organs by dispersing across the insect body *via* hemolymph within the body cavity, which clogs the circulatory system causing the insect’s death. After the host’s death, the fungus enters a facultative feasting stage, when it begins hyphal growth outside the epicuticle and produces many spores (Altinok, Altinok & Koca, 2019).

A soil-borne entomopathogenic fungi (EPF) prefers 20 to 30 °C temperature in agriculture soil for multiplication, morphogenesis, and infections. However, temperature endurance in different strains can vary according to the geographical region. Numerous elements, like copper content and fungicides, might have a detrimental impact on controlling the EPF prevalence in the soil (Uzman et al., 2019; Litwin, Nowak & Rózalska, 2020). Copper is the essential element accumutated in vineyard soils, controlling the growth of the soil borne entomopathogenic fungi which also plays a significant role in controlling microbial infections. Bordeaux and cadmium succinate are major fungicides and their mixture comprised of copper treats soil pathogens and mostly used for controling the growth of fungus in orchard trees (Uzman et al., 2019). In agriculture soil, the EFP genus is dominated by *Metarhizium* spp., rather than *B. bassiana* (Balsamo) (Tkaczuk et al., 2014).

Furthermore, the incidence that leads to occurrence of the specific disease due to microbial or other species over a specific period of time and, EFP did not affect the presence of organic matter for *M. anisopliae* and clay content for *B. bassiana* (Quesada-Moraga, Ruiz-García & Santiago-Álvarez, 2006). Soil-based integrated pest management for fruit flies includes EPF. EPF also helps control flies in agricultural systems. As a result, EPF is an important component of a fruit fly soil-based integrated pest management strategy. Therefore, EFP has become an important strategy for controlling insects or other pests in terms of the soil-based integrated pest management (Sookar, 2013). Compared to other soil microorganisms such as bacteria and protozoa that causes the degradation of organic matter through enzymatic process, these entomopathogenic fungi affect their host by interaction and enter into the body by the epicuticle (Lacey & Shapiro-Ilan, 2008; Tahira, Saeed & Khan, 2014).

Entomopathogenic fungal strains are the common cause of increasing infections in crops, fruits and vegetables thus affecting the large variety of crops worldwide (Sicuia et al., 2014). There is need to control the growth of pathogenic fungi. In this view, the objective of this study is to find the biological control potential of some entomopathogenic fungal strains against the peach fruit fly, *Bactrocera zonata*. As a first and crucial step in developing more effective mycoinsecticides, we screened indigenous Pakistani strains of *M. anisopliae*, *B. bassiana*, and *V. lecanii* for improved activity against *B. zonata* in the laboratory. This comprehensive fungal screening may make more effective control of *B. zonata* in Pakistan.

## Materials and Methods

### Rearing of culture

*B. zonata* was obtained from the Rearing Laboratory of Institute of Plant Protection, Muhammad Nawaz Shareef University of Agriculture, Multan, Punjab, Pakistan. The adults were fed with a mixture of sugar, protein hydrolysate (3:1) and water (El-Sayed, 1979). The eggs produced on a daily basis were collected and raised on their natural hosts, like guava, banana, *etc.* Larvae were dipped in the water after completing the third larval instar and were then collected in fine sand for pupation. The pupa stage was sieved and kept in a screen cage (30 cm × 30 cm × 30 cm) a day before the adult emerged, allowing them to forage, mate, and lay eggs.

#### Preparation procedure for conidiospores

Entomopathogenic fungi inoculants were obtained from the Institute of Soil and Environmental Sciences, University of Agriculture, Faisalabad, Pakistan. Potato dextrose broth was used to cultivate the entomopathogenic fungi. A total of 25 grams of PDB was put into the distilled water and mixed with a magnetic stirrer until all constituents of media were properly dissolved in water. Then PDB media was allowed to pass through the autoclave for 15 min at 121 °C at 15 PSI. In sterilized petri dishes, the autoclaved material was placed then cooled in cleaned laminar flow. At 26 °C temperature (with 75% humidity) PBD carrying Petri plates were incubated for 12 h.

To collect fungal conidium, we lightly stuck the superficial layer of two weeks old colonies by using a sterilized needle. A magnetic shaker was used to immerse it in distilled water and shake it for 10 min. The mixture was then filtered to remove the unwanted elements. The number of fungal spores was counted using a hemocytometer under the microscope. The stock solution was made in sterilized water with the desired concentration of fungal spore (1 × 10^8^ conidia/ml). Gabarty et al. (2014) outlined a series of dilutions that resulted in concentrations of 1 × 10^6^, 1 × 10^7^, 1 × 10^8^, 1 × 10^9^, and 1 × 10^10^ conidia/ml suspensions. These were kept at 4 °C on ice until used in bioassays to inhibit conidial germination.

#### Larval bioassay

Third instar peach fruit fly larvae (*B. zonata*) were evaluated in 7 cm diameter sterilized plastic jars with 75 ml fine sand sieved through a 2 mm sieve. For 60 min at 200 °C the sand and glass jars were sterilized. Conidial concentrations of *M. anisopliae* (1 × 10^6^, 1 × 10^7^, 1 × 10^8^, 1 × 10^9^ and 1 × 10^10^ conidia/ml) were employed and dispersed into the sand using a tiny sprayer and mixing the sand. One-third of the populations of instar were exposed to bio-fungicides. In each jar, they are placed at the surface of treated sand, allowing them to dig into it naturally. Rubber bands and muslin strips were used to close the jars. For each concentration, five replications were performed, and the control was also replicated five times. *B. bassiana* and *V. lecanii* were treated in the same way. The preparations were kept in a laboratory setting at 75% relative humidity and 25 ± 2 °C temperature. After every ten days, the number of emerging adults were counted.

Larvae, pupae, and adult flies were collected and incubated at 25 °C for two days to confirm the fungal infestation. The fungus infestation was confirmed by harvesting killed individuals (larvae, pupa and adult) and was placed into a sterilized Petri-dish with wet cotton and cultured at 25 ± 2 °C temperature until fungal sporulation on the cadaver occurred.

#### Pupal bioassay

Then three-day-old *B. zonata* pupae were laid on the treated sand with different conidial concentrations (1 × 10^6^, 1 × 10^7^, 1 × 10^8^, 1 × 10^9^ and 1 × 10^10^ conidia/ml) after one minute of exposure in conidial suspension. Afterward, the pupae were buried in the rest of the sand (Ekesi, Maniania & Lux, 2002). Each concentration was repeated five times, with a control treatment of five duplicates without conidia. All entomopathogenic fungi were treated in the same way. In each jar, the numbers of newly-emerged flies were recorded. The infection was confirmed by collecting the deceased individuals (pupae) due to a fungus attack. Then the collected individuals were placed into a sterile petri plate with wet cotton and cultured at 25 ± 2 °C until the cadavers produced sporulation.

#### Bioassay test for newly emerged fruit flies

In sterilized jars measuring 23 cm height and 9 cm diameter, the *B. zonata* freshly emerging flies (one day old) were exposed to the entomopathogenic fungus V. lecanii, B. bassiana and M. anisopliae. Fifty newly emerging *B. zonata* adults (one day old) were put into each treated plastic jar with different conidial concentrations (1 × 10^6^, 1 × 10^7^, 1 × 10^8^, 1 × 10^9^ and 1 × 10^10^ conidia/ml) by aspirator, which allowed them to move freely on the surface of jars. The fruit flies were fed an artificial diet while in the jars. We supplied the water, sugar and enzymatic yeast hydrolysate in a (3:1) ratio and covered the jars with muslin for ventilation. Each concentration was repeated five times and a control treatment did not include conidia. V. lecanii and B. bassiana were also treated in the same way. The treatments were kept at a constant temperature of 25 ± 2 °C with a relative humidity of 75%. The infection was confirmed by collecting the killed flies, and then the collected individuals were placed into a sterile petri plate with wet cotton. They incubated at 25 ± 2 °C temperature until on cadavers population appeared.

#### Statistical analysis

Data was anaylsed by applying the ANOVA with HSD and the Abbott formula for accesing the insecticidal efficicay in the form of percentage. The Abbott formula was used to adjust the mortality rate. In entomological field data, the Abbott formula is applied to estimate the mortality rate in different insecticide trials and differentiate the effects of pesticide treatment from those produced by natural variables. The Abbott formula was used to calculate the insecticide efficacy (E) as the following formula:

(1) \begin{eqnarray*}\text{Insecticide efficacy}~\mathrm{E} \left( \text{%} \right) = \frac{T-t}{\mathrm{T}} \times 100.\end{eqnarray*}

In this formula, E is the insecticide efficacy, T is the mean number of alive larvae on control treatment. On the other hand, t is the mean number of alive larvae on each insecticide treatment. To estimate mortality of insects, Probit analysis was performed (Abbott, 1925). Probit analysis is used to examine data from bioassay tests, such as various concentrations of insecticide used to kill proportions of insects. Probit analysis results were often reported as a concentration or time required to kill a specific percentage of test insects (for example, LC_50_) (Finney, 1971). The difference in death rates among treatments was determined using analysis of variance (ANOVA), and Tukey’s method was applied to assess mean significant differences among treatments (*P* < 0.05) using statistical software. SPSS software was used to assess the data.

## Results

### Stability of *B. zonata* larvae to different concentrations of entomopathogenic fungi

The findings revealed that mortality increased considerably with increased conidial spores/ml. At the 1 × 10^6^ and 1 × 10^10^ conidia/ml concentrations, the *B. bassiana* (entomopathogenic fungi) resulted in the greatest percentages of larval mortality, ranging from 48.43 to 68.67%, respectively. Similarly, at the same concentrations as *B. bassiana* and *M. anisopliae* came in second place (38.41–65.33%). On the other hand, *V. lecanii* produced the lowest percentage of larval mortality, ranging from 41.43% to 61.31%, respectively (Table 1).

**Table 1:** Mean percentage of the mortality of *B. zonata* (Saunders) larvae treated with different concentrations of entomopathogenic fungi (*Metarhizium anisopliae* (Met.), *Beauveria bassiana* (Bals.) and *Verticillium lecanii*).

**Concentrations** (Conidia /ml)	**Percent mortality ± S.E.**		
	***M. anisopliae***	***B. bassiana***	***V. lecanii***
1 × 10^6^	38.43^e^± 0.02	48.41^d^± 0.08	41.43^e^± 0.02
1 × 10^7^	43.50^d^± 0.02	54.57^c^± 0.03	47.75^d^± 0.03
1 × 10^8^	49.71^c^± 0.04	59.51^b^± 0.02	59.56^c^± 0.81
1 × 10^9^	57.67^b^± 0.03	61.21^b^± 0.03	58.76^b^± 0.02
1 × 10^10^	65.33^a^± 0.02	68.67^a^± 0.02	61.31^a^± 0.02
Control	0.50^f^± 0.31	0.40^e^± 0.25	0.80^f^± 0.31
*F*	218.00^**^	136.00^**^	119.00^**^
*P*	0.000001		
df	5, 20		

#### Susceptibility of three-day-old pupae of *B. zonata* to different concentrations of entomopathogenic fungi

After exposure to *B. bassiana*, *M. anisopliae* and *V. lecanii*, pupae displayed a range of mortality percent values 89.67%, 85.76%, and 68.67%, respectively. Percentages of mortality were induced by *B. bassiana* and *M. anisopliae* at the 1 × 10^10^ conidial concentration were significant relative to *V. lecanii* (Table 2).

**Table 2:** Mean percentage *B. zonata* (Saunders) three-day-old pupae treated with different concentrations of entomopathogenic fungi (*Metarhizium anisopliae* (Met.), *Beauveria bassiana* (Bals.) and *Verticillium lecanii*).

**Concentrations** (Conidia /ml)	**Percent mortality ± S.E.**		
	***M. anisopliae***	***B. bassiana***	***V. lecanii***
1 × 10^6^	51.43^d^± 0.01	54.51^e^± 0.01	45.34^d^± 0.01
1 × 10^7^	53.67^d^± 0.01	63.33^d^± 0.01	49.88^d^± 0.03
1 × 10^8^	59.33^c^± 0.08	70.56^c^± 0.02	55.67^c^± 0.02
1 × 10^9^	75.53^b^± 0.01	74.67^b^± 0.03	59.34^b^± 0.51
1 × 10^10^	85.76^a^± 0.02	89.67^a^± 0.02	68.67^a^± 0.01
Control	1.03^e^± 0.14	1.60^f^± 0.76	0.98^e^± 0.13
*F*	68.34^**^	237.31^**^	118.53^**^
*P*	0.000001		
Df	5, 20		

#### Susceptibility of *B. zonata* adult flies

All entomopathogenic fungi were most susceptible to adult *B. zonata* flies. *M. anisopliae* was more virulent than *B. bassiana* and *V. lecanii* at 1 × 10^10^ conidial concentration (Table 3). The results showed that the lower LC_50_ and LC_90_ values for *B. zonata* larvae were recorded by exposed *B. bassiana* which less than values for larvae treated with *M. anisopliae* and *V. lecanii*, with 4.31 × 10^5^ and 1.57 × 10^10^, 1.41 × 10^7^ and 1.95 × 10^11^, 1.23 × 10^7^ and 1.81 × 10^11^ conidia/ml, respectively. Interestingly, *B. bassiana* LC_50_ and LC_90_ were higher toxicity in the treated pupal stage as compared to *V. lecanii* and *M. anisopliae* with values of 1.56 × 10^5^ and 1.32 × 10^9^, 3.45 × 10^5^, 1.66 × 10^15^, 2.01 × 10^5^, 1.63 × 10^11^ conidia/ml, respectively. The results indicated that *B. zonata* flies respond favorably to both *V. lecanii* and *B. bassiana* as compared to *M. anisopliae* with LC_50_ and LC_90_ values of 1.34 × 10^6^ and 5.21 × 10^9^ and 1.023 × 10^6^ and 3.13 × 10^9^ conidia/ml and *M. anisopliae* 2.11 × 10^6^ and 5.21 × 10^9^, respectively (Table 4). Our result showed that *B. bassiana* have higher toxicity against pupal stage depending on LC_50_ and LC_90_.

**Table 3:** Mean percentage mortality of *B. zonata* (Saunders) adult treated with different concentrations of entomopathogenic fungi (*Metarhizium anisopliae* (Met.), *Beauveria bassiana* (Bals.) and *Verticillium lecanii*).

**Concentrations** (Conidia /ml)	**Percent mortality ± S.E.**		
	***M. anisopliae***	***B. bassiana***	***V. lecanii***
1 × 10^6^	42.32^d^± 0.04	45.76^e^± 0.01	39.67^d^± 0.02
1 × 10^7^	48.56^d^± 0.02	54.56^d^± 0.03	47.54^d^± 0.08
1 × 10^8^	61.57^c^± 0.91	59.34^c^± 0.08	55.67^c^± 0.02
1 × 10^9^	69.43^b^± 0.03	72.75^b^± 0.01	62.43^b^± 0.41
1 × 10^10^	86.89^a^± 0.08	84.34^a^± 0.12	74.89^a^± 0.01
Control	0.00^e^± 0.00	0.00^f^± 0.00	0.76^e^± 0.03
*F*	128.51^**^	248.34^**^	78.20^**^
*P*	0.000001		
Df	5, 20		

**Table 4:** Toxicity of *Metarhizium anisopliae* (Met.), *Verticillium lecanii* and *Beauveria bassiana* (Bals.) on various life stages of *B. zonata* (Bals.).

**Stage**		**LC**_50_ **conidia/ml**	**LC**_90_ **Conidia/ml**	**Slope ± SE**	*χ* ^2^	***P***
*M. anisopliae*	Third Instar larvae	1.41 × 10^7^	1.95 × 10^11^	0.206 ± 0.565	0.032	0.7893
	Three Day Old Pupae	2.01 × 10^5^	1.63 × 10^11^	0.029 ± 0.057	0.176	0.5967
	Adult flies	2.11 × 10^6^	5.21 × 10^9^	0.521 ± 0.056	3.562	0.369
*B. bassiana*	Third Instar larvae	4.31 × 10^5^	1.57 × 10^10^	0.273 ± 0.063	0.923	0.8531
	Three Day Old Pupae	1.56 × 10^5^	1.32 × 10^9^	0.354 ± 0.071	1.235	0.9472
	Adult flies	1.023 × 10^6^	3.13 × 10^9^	0.576 ± 0.032	0.564	0.764
*V. lecanii*	Third Instar larvae	1.23 × 10^7^	1.81 × 10^11^	0.315 ± 0.853	0.041	0.8974
	Three Day Old Pupae	3.45 × 10^5^	1.66 × 10^15^	0.107 ± 0.074	0.546	0.9829
	Adult flies	1.34 × 10^6^	5.21 × 10^9^	0.749 ± 0.067	3.473	0.153

**Figure 1:**
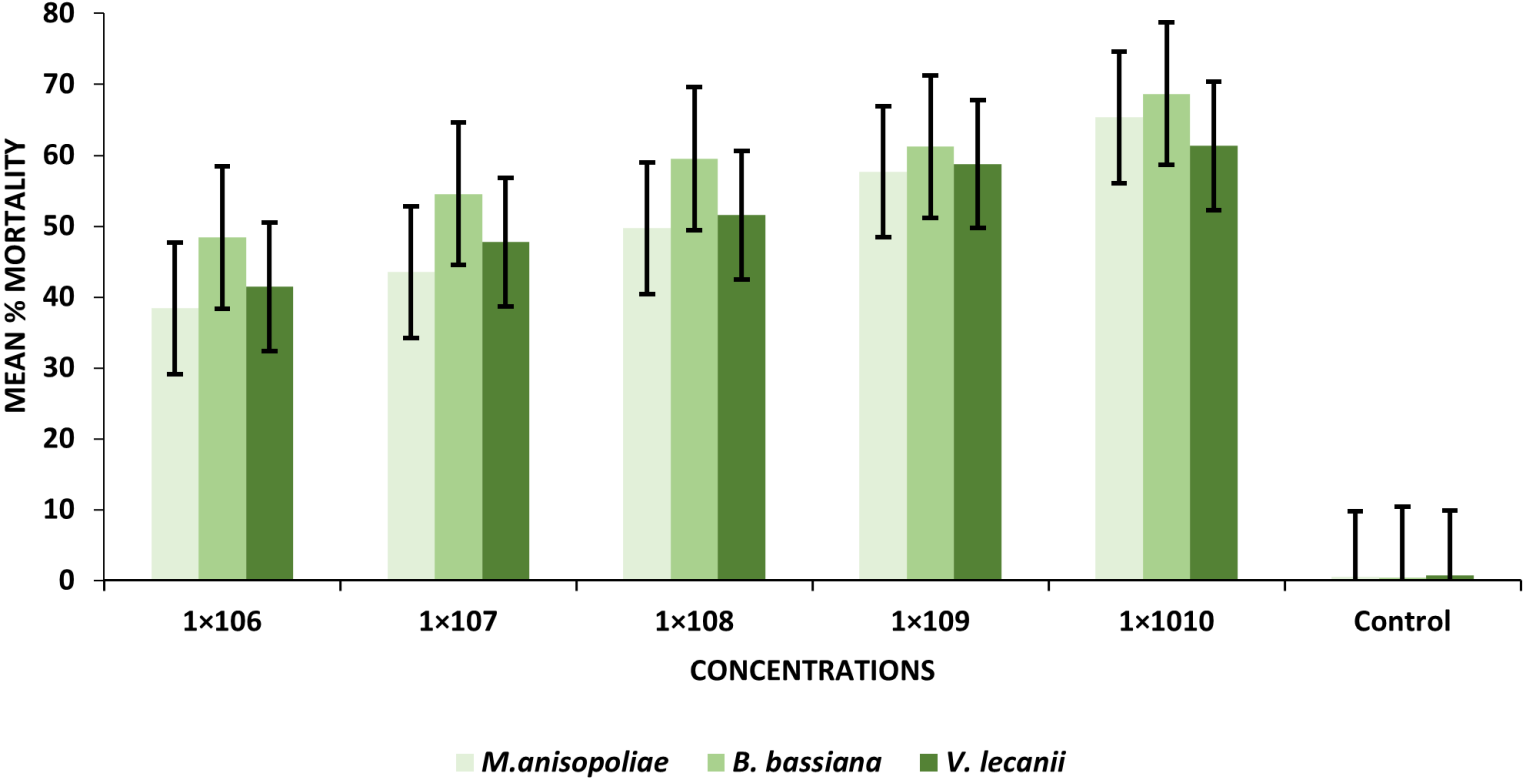
Percentage mortality of *B. zonata* (Saunders) larvae with all entomopathogenic fungi.

**Figure 2:**
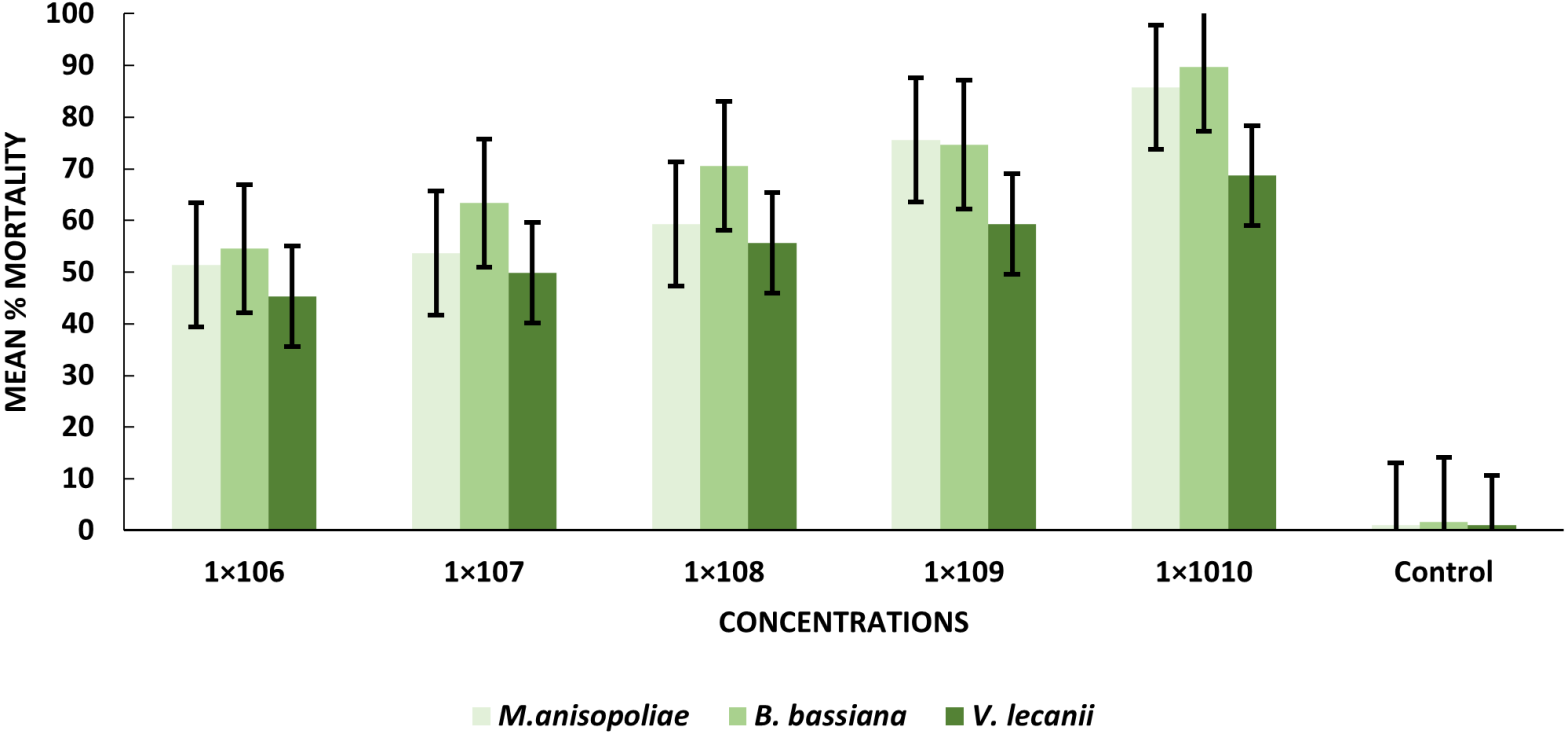
Percentage mortality of *B. zonata* (Saunders) pupae with all entomopathogenic fungi.

**Figure 3:**
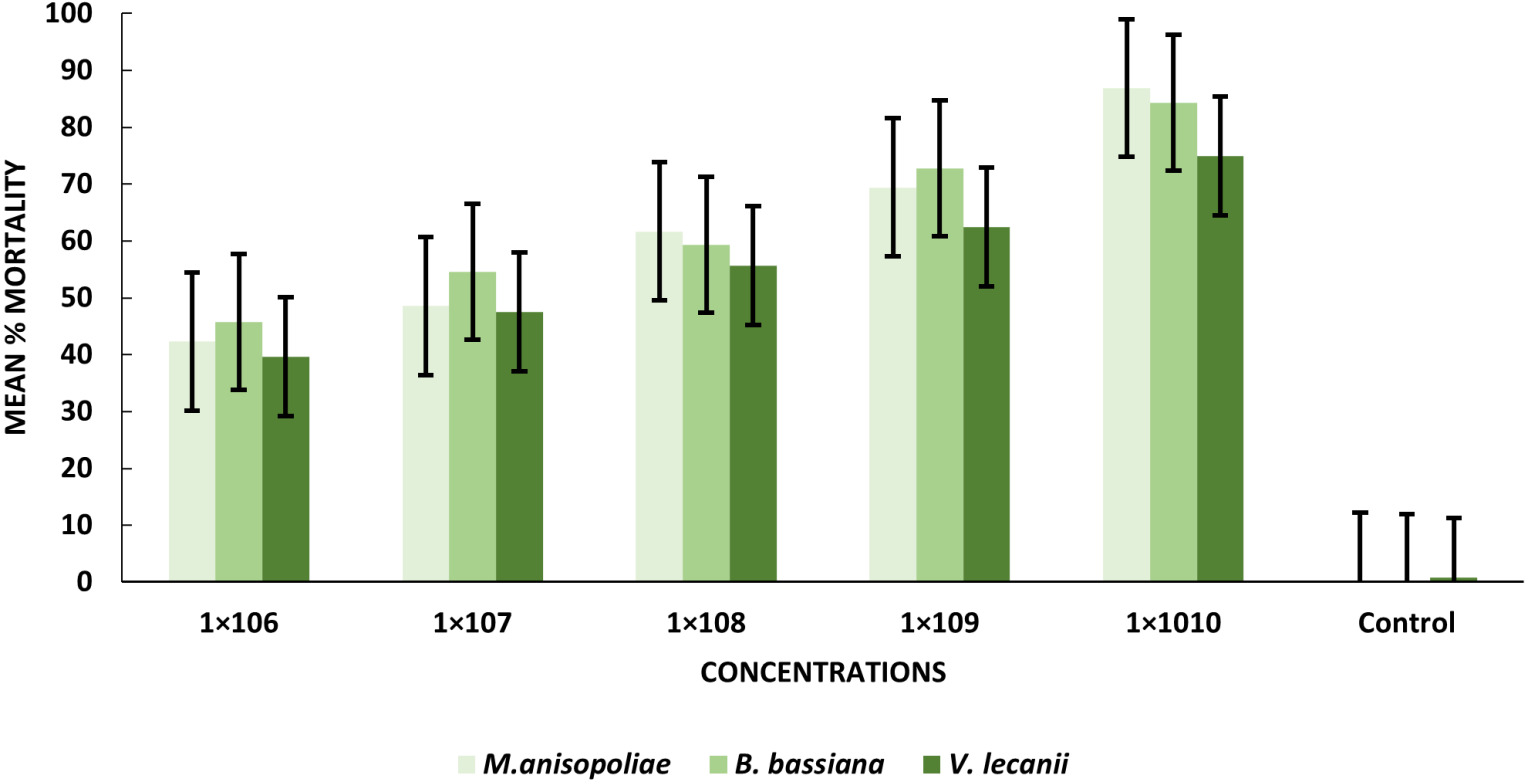
Percentage mortality of *B. zonata* (Saunders) adult fly with all entomopathogenic fungi.

The statistical analysis was used to compare the efficacy of the tested fungi against larval instar at the medium 1 × 10^8^ and maximum 1 ×10^10^ concentrations. The obtained results demonstrated that *B. bassiana* had a higher ability to kill *B. zonata* larvae than *M. anisopliae* and *V. lecanii* (Fig. 1). The differences in mortality between EPF at the same dose were statistically examined to identify which of the tested fungus could kill pupal stage specimens the most effectively. The obtained findings showed that *M. anisopliae* and *B. bassiana* were more virulent to kill three-day old pupae at medium 1 × 10^8^ and maximum concentration 1 × 10^10^ than *V. lecanii* (Fig. 2). The variations in mortality between EPF at the medium 1 × 10^8^ and maximum 1 × 10^10^ concentrations were statistically evaluated to determine which of the tested fungus have more potential to kill the adult stage. The obtained results demonstrated that *M. anisopliae* and *B. bassiana* were more virulent than *V. lecanii* kill adult flies (Fig. 3).

## Discussion

Pesticides like malathion, whether used aerially or on the ground, can severely influence the ecosystem, including the killing of non-target species. It is highly toxic for insects and commonly used for controlling the growth of insects. It binds to the nerve endings of the insects and caused the neurological damage by blocking the acetyl cholinesterase (Peck & McQuate, 2000; Leach & Mumford, 2008). Use of more environmentally friendly bait sprays, such as spinosad, may be an alternative for preventing or reducing these negative consequences (Burns et al., 2001; Chueca et al., 2007; Jesus et al., 2009). Protected areas, urban and suburban regions, lake basins and ecological reserves are excluded from area-wide pest control programs that employ these ecologically friendly substances (Vargas et al., 2008). Microbial control (MC) using entomopathogenic fungus is an ecologically favorable, human innocuous, and long-term pest control strategy (Zimmermann, 2007). Entomopathogenic fungi are a viable pest management strategy in organic and conventional farming systems (Lomer et al., 2001; Meyling & Eilenberg, 2007; Flores et al., 2013).

According to the application technique, the pathogen’s viability and transmission capabilities define the pathogen’s range of spread in the host (Vega et al., 2000; Toledo et al., 2007; Quesada-Moraga et al., 2008). Research on viability and pathogenicity of entomopathogenic fungi such as *M. anisopliae*, *B. bassiana* and *V. lecanii* and how to apply them in the field has supported the development of MC technology for fruit flies.

Our results agreed with the previous studies that *B. bassiana* had a higher ability to kill *B. zonata* larvae than *M. anisopliae* and *V. lecanii* (Hussein et al., 2018; Soliman et al., 2020). The cuticle is the main channel of fungal penetration in insects, and pupal susceptibility decreases as the pupal age increases (Ekesi, Maniania & Lux, 2002; Hussein et al., 2018). Different physical and enzymatic methods are required to control the EPF for adopting the agricultural strategy in such conditions (Tahira, Saeed & Khan, 2014). The findings showed that *M. anisopliae* and *B. bassiana* were more virulent to kill three-day-old pupae at medium 1 × 10^8^ and maximum concentration 1 × 10^10^ (Hussein et al., 2018; Soliman et al., 2020) as compared to *V. lecanii*.

Many scientists agree with these findings and report that adult fruit flies were more sensitive to various EPF. Temperature and humidity are very important environmental factors not only affecting the efficacy but also the survival of an entomopathogen (Zimmermann, 2007). Our results demonstrate that *B. bassiana* and *M. anisopliae* were more virulent than *V. lecanii* kill adult flies (Daniel & Wyss, 2008; Daniel, 2009; Hussein et al., 2018; Soliman et al., 2020). In comparison to the control treatment, significant mortality was observed in all entomopathogenic fungi. The findings of this study agree with various researchers (Oliveira et al., 2010; Ghulam Ali et al., 2018; Hussein et al., 2018), who found that increasing *B. bassiana* and *M. anisopliae* conidia concentrations increased the mortality of larvae and pupae of the fruit fly.

Entomopathogenic fungi generate a variety of poisons and enzymatic activities such as chitinases and proteases when interacting with the initial hosts. Toxicity values of the used entomopathogenic fungus based on LC^50^ and LC^90^ indicated the virulence of *B. bassiana* to kill all *B. zonat* more than *M. anisopliae* and *V. lecanii*. (Castillo et al., 2000) reported that *Verticillium lecanii* showed low mortalities (>10%) against *C. capitata, M. anisopliae, B. bassiana* also showed high virulence 92 and 80% mortality rates against *B. oleae*, respectively. Laboratories tests demonstrated that fungal species (bio-pesticides) have a higher capacity to prevent pest loss (Daniel & Wyss, 2008; Ladurner et al., 2008; Daniel, 2009). Furthermore, alternative strains might be formulated for mass manufacturing to be employed as wide-spectrum mycopesticides.

## Conclusion

In conclusion, these findings marked a significant step in the development of ecologically friendly natural biocontrol entomopathogenic fungi to control the invasive fruit fly pest. In addition, current findings demonstrated that *B. bassiana* was the most lethal and virulent strain to the targeted larvae and adult flies compared to *M. anisopliae* and *V. lecanii*. While *V. lecanii* were more toxic at the adult stage than *M. anisopliae*, both fungi could potentially contribute to mitigating the burden of this pest and minimizing any likelihood of host resistance to a high-virulence strain. Based on evidence, we may infer that entomopathogenic fungi are harmless and pose few hazards. Concerning the commercialization and registration of future isolates, the question is what further rules and testing are required to provide the user and customer with a safe biocontrol product. This study will be helpful to overcome this invasive pest through the biological method, and in the future, we need to study and explore comprehensive methods to control quarantine pests.

## Supplemental Information

10.7717/peerj.13316/supp-1 Data S1 Data for the graphical presentation of life stages of Bactrocera zonata (Saunders) with the effect of different fungi strains treatments

10.7717/peerj.13316/supp-2 Data S2 Raw data collected during the experimentation for the treatment and control groups and later used for statistical analysis

## Additional Information and Declarations

### Data Availability

The following information was supplied regarding data availability: The raw data is available in the Supplemental Files.
